# Assessing the Impact of A Community-Based Pro-Active Monitoring Program Addressing the need for Care of Community-Dwelling Citizens aged more than 80: Protocol for a Prospective Pragmatic Trial and Results of the Baseline Assessment

**DOI:** 10.37825/2239-9747.1004

**Published:** 2020-10-01

**Authors:** G Liotta, O Madaro, P Scarcella, MC Inzerilli, B Frattini, F Riccardi, N Accarino, S Mancinelli, E Terracciano, S Orlando, MC Marazzi

**Affiliations:** 1 Department of Biomedicine and Prevention, University of Rome “Tor Vergata”, Via Montpellier 1, 00173, Rome, Italy; 2 Community of Sant’Egidio, “Long Live the Elderly!” program, Via San Gallicano 25, 00153, Rome, Italy; 3 LUMSA University, Via della Traspontina 21, 00193, Rome, Italy

**Keywords:** bio-psycho-social frailty, Functional Geriatric Evaluation, hospital admission rate, mortality, social isolation

## Abstract

The aim of this paper is to describe the protocol of a study assessing the impact of a Community-based pro-Active Monitoring Program, by measuring the effect in counteracting the adverse outcomes related to frailty.

**Methods:**

a prospective pragmatic trial will be carried out to describe the impact of an intervention on people aged>80, adjusted for relevant parameters: demographic variables, comorbidities, disability and bio-psycho-social frailty. They have been assessed with the Functional Geriatric Evaluation questionnaire that is a validated tool. Mortality, Acute Hospital Admission rates, Emergency Room Visit rates and Institutionalization rates are the main outcomes to be evaluated annually, over three years. Two groups of patients, made up by 578 cases (undergoing the intervention under study) and 607 controls have been enrolled and interviewed.

**Results:**

at baseline the two groups are quite similar for age, living arrangement, comorbidity, disability and cognitive status. They differ in education, economic resources and physical status (that are better in the control group) and in social resources (that is better in the case group). The latter was expected since the intervention is focused on increasing social capital at individual and community level and aimed at improving survival among the cases as well as reducing the recourse to hospital and residential Long Term Care.

**Conclusion:**

The proposed study addresses a crucial issue: assessing the impact of a *bottom up care* service consisting of social and health interventions aimed at reducing social isolation and improving access to health care services.

## I. INTRODUCTION

The implementation of effective community care services for older adults with disability or at risk of disability is a crucial point for improving older citizens quality of life and providing appropriate care at affordable costs [1]. In order to reach this objective, the stratification of older population according to risk of negative events (i.e functional status worsening, admission to hospital or to Long Term Care (LTC) residential facilities, death) and to amount of care demand is needed [2,3].

The most effective synthetic indicator of these two factors is bio-psycho-social frailty, that can be assessed with several validated instruments [4,5]. In fact, bio-psychosocial frailty is a multidimensional reversible condition predisposing to functional decline in older adults [6,7]. The assessment of frailty is associated to the risk of negative events as well as to the amount of the demand for care and it can address towards the most effective intervention. In fact, the frailty status is associated to a more urgent care demand, addressed mainly to LTC services, while the prefrail status could be effectively managed by prevention practices and active monitoring [8,9].

“Long Live the Elderly!” (LLE) is a Community-based pro-Active Monitoring Program (CAMP) born in 2003 to fight social isolation that is a risk factor for adverse events among older adults [10]. Social isolation represents an aspect of older adults frailty and it is related to the extension and quality of the individual’s relationship network. It is associated to higher risk of death, hospitalization and institutionalization [11]. During the 2003 summer a heat wave hit Southern Europe provoking about 20,000 of unexpected deaths, mainly among citizens over-74 living alone [12]. The LLE program is directed to over-74 years old citizens with a special focus on the over-80s because frailty is three folds higher among the over-74 compared with the 65 – 74 age group.

The general aim of the LLE program is to increase the social capital of both the community and the individual. The program provides phone monitoring to all the clients and home visits according to the individual’s needs. Moreover, it activates other formal or informal care resources according to the patients need reported in the Individualized Care Plan (ICP) which stems from the assessment of multidimensional frailty. The operators of the program are holders of at least a secondary school diploma and trained ad hoc for performing CAMP intervention. The main peculiarity of the program is that the operators identify the main problem of the client and try to track down the better solution in agreement with the client itself. It can be a health or social or a different kind intervention. Interventions may include the assistance to make safe the clients’ house thereby reducing risk factors for falls or revising the therapeutic scheme to improve the patient’s adherence to the treatment in collaboration with the GP. It is a *bottom up approach* [13] to overcome the separation between health and social care, that is still a burning issue at community care level in Italy as in many European countries. Some evidence seems to confirm the positive impact of the program on mortality, hospitalization and institutionalization [14,15]. The LLE program is operating in several Italian cities keeping on charge about 14,000 over-75 citizens. Aim of this paper is to describe the protocol of a study assessing the impact of a Community-based pro-Active Monitoring Program, on the quality of life and survival of people aged>80. The paper also provides information on baseline characteristics of the sample enrolled in the study.

## II. METHODOLOGY

The study is designed as a pragmatic trial comparing two groups of over-80s: the first one has been randomized among the LLE clients in two cities: Rome and Naples: the randomization has been performed on the LLE central database that includes all the participants to the program in Naples and Rome who have been administered the Functional Geriatric Evaluation (FGE) questionnaire [16,17], (3358 and 904 aged>80 people for Rome and Naples respectively). The entry point of the study is the administration of the FGE questionnaire. Periodical follow-ups are included in the program. The control group is selected by randomization from a pool of over-80s followed up by General Practitioners in the same cities who have been available to be involved in the study. Each GP provided a list of patients which 10 names have been selected from by randomization. The total pool consisted of approximately 8500 individuals. The sample was made up by 690 selected patients of which 83 (12.02%) refused to participate to the study.

The study has been approved by the Indipendent Ethical Committee of the University of Rome “Tor Vergata “ (R.S. 60/17). Participants gave their written consensus to participate to the study

### Inclusion and exclusion criteria

People enrolled into the study must be older than 80 years old and had to answer to the FGE questionnaire. People living in an institution (nursing homes or similar) have been excluded. Advanced mental impairment was not an exclusion criteria, but in such cases the consent have been signed by the closest relative who also answered to the questionnaire on behalf of the participant; this modality is foreseen by the extensors of the questionnaire, just for these cases. The selected patients have been contacted by phone and they underwent a face-to-face interview by trained personnel in the GP’s outpatient facility or at home if they were unable to go out.

### Sample size

The primary outcome is the difference in hospitalization and mortality rate between the LLE sample and the controls. Based on previous analyses, the three years expected hospital admission rate and death rate for the over-80 population accessing the standard of care are 35% and 25%, respectively. The maximum foreseen incidence rates in the population undergoing the LLE program intervention are 25% and 18% for hospitalization and death, respectively. The needed sample size in this case is 540 subjects per arm (Alpha error = 5%, Beta error = 20%). Based on the number of over-80s residents in Rome and Naples, a total sample of 1080 individuals is enough to assess differences in incidence rate per person/year higher than ±3%; a sample size of 600 individuals per each city is enough to assess differences in incidence rate higher than ±4%.

### Baseline assessment

The FGE questionnaire provides a multidimensional assessment and allows a definition of frailty using a final score [14,17]. The FGE has been validated by the researchers of Biomedicine and Prevention Department of University of Rome “Tor Vergata” as predictor of death, hospital use and need of LTC, and used in several studies. It consists of four sections:

- Demographic information
- Multidimensional evaluation (physical, mental and functional status, socio-economic resources, environment): a score is given to each domain of this section and contributes to the Final Score. in each domain, as it is for the Final Score, the higher is the score the better the client’s situation
- A list of diseases affecting the patients, compiled by the GPs
- Activities of Daily Life (ADL) according to Katz and Instrumental Activities of Daily Life (IADL) according to Lawton.

The Multidimensional evaluation (section b) contributes to generate a Final Score, that ranges from −108 to 101 while the other information are used as independent variables. According to the Final Synthetic Score (FSS) the subjects are classified in 4 groups: Robust : FSS >70; Pre-frail: FSS 50–70; Frail: FSS 11–49; Very Frail: FSS <10

### Follow up data collection

Follow up data will be gathered every six months over a period of three years. Data will be collected through:

- Phone interviews to enrolled subjects
- Phone interviews to enrolled subjects’ GPs
- Information gathered from Regional data base on:
  ○ Hospital admissions and Emergency Room (ER) accesses
  ○ Mortality
  ○ Admissions to LTC facilities
  ○ Use of home care services

### Outcomes

The following outcomes will be assessed for each site and each group :

- Incidence of hospitalization and ER access
- Incidence of death
- Incidence of admission to LTC facilities
- Lost to Follow Up (LTFU)

### Statistical Methods

Continuous and categorical variables have been displayed; differences between the two groups have been tested by parametric and non parametric tests. The statistical analysis was performed through IBM SPSS Statistics 25.0,

## III. RESULTS

The sample was made up by 1,185 individuals, 578 included in the LLE program and 607 controls (Table 1).

Mean age was quite similar between the two groups (84.8±5.7 and 83.7±4.8 for LLE and controls, respectively) even if the difference is statistically significant (U-Mann-Withney Test; p<0.001). However, Table 2 shows baseline characteristics and some significant statistic differences: female gender is less represented among the controls as well as the older age group (people> 85 years old are 43.2% vs 31.0% among cases and controls respectively). Controls are also more educated while living arrangements do not show statistic significant differences. Comorbidity is more prevalent among the controls (92.7% vs 87.3%) even if the median of the number of pathologies is 4 for the controls and 5 for LLE group (p = 0.025).

**Fig 1:**
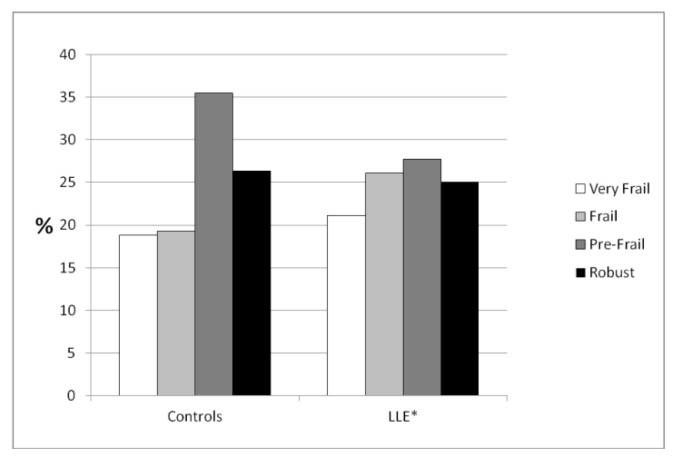
Level of frailty *“Long Live the Elderly!” program

The assessment of frailty shows that the control group is less likely to be frail than the LLE group: overall frail and very frail individuals are less than 40% in the control group while the percentage is close to 50% among the LLE group (Chi-square test; p=0.005) (Fig 1).

With regard to the single domain of the assessment of frailty (Table 3), the control group shows a better Physical Area Score (−10.5 vs −13.7, p<0.001) and a better Economic Area score (9.8 vs 8.6, p<0.001). However, the LLE group shows a better score in the Social Area (27.4 vs 22.0, p<0.001).

Interestingly, comorbidity was correlated to each Area score with statistic significance (Pearson correlation: p<0.01 for each score) as well as with FGE Final Score, but it did not correlate with age.

## IV. DISCUSSION

The paper reports on the design of a longitudinal pragmatic trial aimed at evaluating the impact of a community care intervention based on the assessment of frailty and on counteracting social isolation in two Italian cities with a low rate of community care services. The sample is made up by two population of over-80s individuals: the first one accessing the standard of care and the second one included in the LLE program. The differences between the two samples are due to the trial design aimed at comparing populations who underwent different care interventions. In this case, the differences reported by the paper in physical or social areas score are crucial to assess any gap among the outcomes. Bio-psycho-social frailty is associated to an increase of mortality and use of hospital services; social isolation is considered a major risk factor for developing frailty[18–20] as well as specific diseases associated to advanced frailty (like dementia) [21], especially in the older adult population. It is likely that a program focused on counteracting social isolation is able to slow down or even reverse the progression towards frailty and reduce the incidence of negative events [12, 14, 22]. Some evidence is already documented; however, to our knowledge, it is the first time that such a program is tested in a pragmatic trial at community level.

The effectiveness of intervention aimed to counteract loneliness or social isolation has been discussed since many years ago [23]. The questions raised are often methodological, but there is also another issue: most of the studied interventions are addressing strictly social problems, without considering the inextricable intertwining between social and health issues. In many cases, group interventions have been considered more effective than interventions targeting the individual in his/her own living environment. However, even in this case the impact on the citizens’ health and on their use of health care services were not assessed [24].

Fairhall and coll. performed a randomized clinical trial on a sample of individuals assessed for physical frailty according to the Fried criteria: they tested an intervention aimed at improving physical performance of part of the sample to be compared with the other ones who accessed the standard of care. They pointed out the positive impact of the intervention, especially for males, and for “very frail” subjects i.e. participants who met >3 Cardiovascular Health Study frailty criteria. In this case, the assessed intervention was trying to improve the patients’ health status, starting from an assessment of patients’ functional health [25,26].

In our case, we are going to test a different intervention based on the provision of social and health integrated care: frail participants will be supported through an ICP drafted by the social worker and the community nurse (when available) or by other professionals like the GP according to the needs of the client and the availability of other professionals to be involved in the program. The assessment of strong outcome indicators like mortality and use of hospital and non-hospital care services strengthens the analysis from a public health point of view [27], and it is in line with the hypothesis to be tested: social intervention are able to improve health and quality of life of participants. The program acts as a case manager, able to involve formal and informal care givers, like relatives or neighbours, whose availability has been previously asked on a voluntary basis or who are operating on the field (as the home care services by municipality or by regional health system).

The two groups show some differences as it occurs in a real world setting: in fact the two samples stems from the Community-based pro-Active Monitoring Program “Long Live the Elderly!” that is an ordinary service ongoing since 2004,, compared with an a cohort set up for this comparison. The main differences are about the physical condition area score, that is worse in the LLE group which at the same time shows higher social resources: this could be understandable since the LLE program is aimed at increasing the social capital at both individual and community level. We are probably witnessing one of the impacts of the program. It is also likely that individuals with advanced physical impairment can stay at home in case of the presence of a supportive social environment able to provide a certain amount of simple daily care. This is even more interesting because the LLE group shows a lower education level which is a proxy of the individual social and economic background with no great differences in the living arrangements. Cognitive and Functional area score have no significant differences between the two groups. It appears that the condition strictly associated to social background of the individuals are worse in the LLE group, so that the better score in the Social Area is due to other factors related to social relationships or to the intervention of formal care services catalyzed by the program.

## V. CONCLUSION

,Social factors are increasing their relevance as determinants of negative health outcomes and increased care demand in an aged society. The need for evidence supporting public health policy investing in integrated health and social services is urgent. Available evidence is mainly referred to services addressing separately social and health care. The paper presents the design of a pragmatic trial to assess the impact of an integrated health and social intervention delivered at community level. Moreover, it is aimed at counteracting social isolation with its negative consequences on health and on demand for care of the older adults

## Figures and Tables

**Table 1:** the sample in the two cities involved

		LLE	Controls	Total
**Cities**	**Napoli**	250	203	453
	**Roma**	328	404	732
**Total**		578	607	1185

**Table 2:** Baseline characteristics

		LLE * (%)	Controls (%)	Pearson Chi^2^
**Town**	**Rome**	44.8	55.2	0.01
	**Napoli**	55.2	44.8	
**Gender**	**Females**	68.7	58.5	<0.001
	**Males**	30.3	41.5	
**Age groups**	**<85**	56.8	69.0	<0.001
	**>85**	43.2	31.0	
**Education**	**Primary School**	78.2	67.2	<0.001
	**Secondary School/Degree**	21.8	32.8	
**Living arrangements**	**Alone**	15.9	18.3	0.536
	**Spouse**	59.7	58.6	
	**Other**	24.4	23.1	
**Comorbidity (more than one disease)**		87.3	92.7	<0.001

**Table 3:** Mean Score per domain

	LLE	Controls	U Mann-Withney Test
			p
**Physical Area score**	−13.7 (SD ±13.8)	−10.5 (SD ±12.4)	<0.001
**Cognitive Area score**	−7.9 (SD±14.0)	−6.8 (SD±12.8)	NS
**Functional Area score**	28.6 (SD ±14.5)	29.5 (SD ±14.8)	NS
**Social Area score**	27.4 (SD ±5.9)	22.0 (SD ±6.1)	<0.001
**Economic Area score**	8.6 (SD±4.8)	9.8 (SD±5.5)	<0.001
